# Immunometabolism of Myeloid-Derived Suppressor Cells: Implications for *Mycobacterium tuberculosis* Infection and Insights from Tumor Biology

**DOI:** 10.3390/ijms23073512

**Published:** 2022-03-23

**Authors:** Brian S. M. Munansangu, Colin Kenyon, Gerhard Walzl, André G. Loxton, Leigh A. Kotze, Nelita du Plessis

**Affiliations:** DSI-NRF Centre of Excellence for Biomedical Tuberculosis Research, South African Medical Research Council Centre for Tuberculosis Research, Division of Molecular Biology and Human Genetics, Faculty of Medicine and Health Sciences, Stellenbosch University, Cape Town 7505, South Africa; munansangu@sun.ac.za (B.S.M.M.); kenyon@sun.ac.za (C.K.); gwalzl@sun.ac.za (G.W.); gl2@sun.ac.za (A.G.L.); lakotze@sun.ac.za (L.A.K.)

**Keywords:** MDSC, tuberculosis, metabolic reprogramming, glycolysis, OXPHOS, immunometabolism

## Abstract

The field of immunometabolism seeks to decipher the complex interplay between the immune system and the associated metabolic pathways. The role of small molecules that can target specific metabolic pathways and subsequently alter the immune landscape provides a desirable platform for new therapeutic interventions. Immunotherapeutic targeting of suppressive cell populations, such as myeloid-derived suppressor cells (MDSC), by small molecules has shown promise in pathologies such as cancer and support testing of similar host-directed therapeutic approaches in MDSC-inducing conditions such as tuberculosis (TB). MDSC exhibit a remarkable ability to suppress T-cell responses in those with TB disease. In tumors, MDSC exhibit considerable plasticity and can undergo metabolic reprogramming from glycolysis to fatty acid oxidation (FAO) and oxidative phosphorylation (OXPHOS) to facilitate their immunosuppressive functions. In this review we look at the role of MDSC during M. tb infection and how their metabolic reprogramming aids in the exacerbation of active disease and highlight the possible MDSC-targeted metabolic pathways utilized during M. tb infection, suggesting ways to manipulate these cells in search of novel insights for anti-TB therapies.

## 1. Introduction

Myeloid-derived suppressor cells (MDSC) have been identified and described in various solid tumor microenvironments to facilitate malignant growth and metastasis, contributing to refractory tumors [1,2,3,4,5]. Evidence for direct correlation between tumor burden and MDSC frequency in mouse tumor models and clinical studies has also been reported [6,7,8]. MDSC have also been observed in other pathologies such as autoimmune [9], viral [10], and infectious diseases [11]. The current outbreak of the novel severe acute respiratory syndrome coronavirus 2 (SARS-CoV-2), the etiological agent for the coronavirus disease (COVID-19), provided more evidence of the detrimental regulatory role of MDSC in many infectious diseases [12,13]. Studies of how SARS-CoV-2 alters intracellular metabolism especially of innate cells during infection have found some intriguing biochemical aspects, including increased glycolysis and dysregulation OXPHOS [14].

*Mycobacterium tuberculosis* (M. tb) pathogenesis is usually thought to initially infect alveolar macrophages (AM), leading to recruitment of other inflammatory myeloid cells and subsequent formation of a mature granuloma [15,16]. Over time, the granuloma can disintegrate, releasing the bacilli, which can alter oxygen supply and decrease nutrient availability in the microenvironment [16].

Mounting evidence points to MDSC participation also in bacterial infections such as those propagated by Gram-negative and Gram-positive bacteria [17,18]. Our work in M. tb infection has shown an increased frequency of innate MDSC during active TB, with elevated to similar levels as those observed in cancer patients, including higher frequencies in healthy persons recently exposed to M. tb [19]. These findings raised the hypothesis that MDSC induced during M. tb infection could help indicate disease progression and/or persistence in the host since successful anti-TB medication led to the return to normal frequency of MDSC levels [19]. Considering that the immunometabolic profile of cells influences their activation and function, a thorough understanding of MDSC metabolism is crucial to recognize how these cells might shape immunity to M. tb, specifically in the granuloma. Given the potentially important role of MDSC during M. tb infection, the immune metabolic state of MDSC in the context of TB will be discussed in this review.

## 2. MDSC Classification

### 2.1. Origin and Identification

MDSC originate in the bone marrow during the development of myeloid progenitor cells, i.e., monocytes and neutrophils [20]. MDSC are divided into two major populations in both humans and mice, namely, polymorphonuclear MDSC (PMN-MDSC) and monocytic MDSC (M-MDSC), as described previously in humans [21,22] and mice [23,24]. Recently, the markers lectin-type oxidized LDL receptor 1 (LOX1) [25] and fatty acid transporter 2 (FATP2) [26] have been shown to discriminate MDSC in tumor biology, although these have not yet been confirmed to be expressed by MDSC in infectious diseases such as active TB disease. Although M-MDSC and PMN-MDSC are considered the two main subsets of this suppressive population, recent evidence from human studies points towards a precursor subset termed early-stage MDSC [27]. Agrawal et al. also described a subverted DC phenotype characterized by low expression of MHC class II and CD80, and expression of CD14 that lacked CD1a molecule with the presence of CD83 and CD86 [28]. Additionally, our research group has also shown that CD14^+^ MDSC produce IL-10 and Il-6 with low levels of HLA-DR and CD80 [28,29]. Another recently described subset of MDSC has been termed eosinophilic (Eo)-MDSC and was identified in a mouse model infected with *Staphylococcus aureus* [30].

### 2.2. Recruitment and Expansion of MDSC

MDSC are recruited from the bone marrow in response to a variety of growth factors, hormones, and transcription factors, along with antigens linked to these pro-inflammatory conditions, thereby regulating myelopoiesis and inducing MDSC expansion. This process is enhanced by alarmins such as prostaglandin E2 (PGE2) and high mobility box group 1 (HMGB1) secreted by host tissues [31,32], and pro-inflammatory cytokines such as tumor necrosis factor (TNF-α), interleukin-1β (IL-1β), IL-6, IL-13, and S100A8/A9 [33]. Hypoxia and nutrient starvation may also affect the epigenetic status of myeloid cells [34], which is critical to the accumulation and reprogramming of MDSC in response to inflammatory or pathogenic insults [35]. Additionally, chemokines such as CCL2 (MCP-1, monocyte chemoattractant protein-1), CXCL2 (MIP-2, macrophage inflammatory protein 2), and CXCL8 have been identified to be vital in MDSC migration [36,37].

### 2.3. Immunosuppressive Mechanisms of MDSC

Although MDSC are widely considered regulators of the immune response, contributing to the homeostatic balance between pro- and anti-inflammatory responses, under aberrant conditions of chronic infection, MDSC are mainly considered detrimental to efficient host control of the pathogen. Several studies have shown how metabolic alterations, as well as those induced by infections, can influence the function, reprogramming, and differentiation of MDSC [38]. The mechanisms by which these cells suppress the immune response include:

(i) Sequestration of cardinal amino acids (AA) such as L-arginine, L-cysteine (Cys), and L-tryptophan by the activity of inducible enzymes such as arginase 1 (ARG1), inducible nitric oxide synthase (iNOS/NOS2) and indolamine dioxygenase (IDO) [27,39]. The reduction of these AA leads to the inhibition of T-cell activation and proliferation and reduced expression of T-cell receptor TCR-CD3 ζ chain, inducing the cell to undergo proliferative arrest [40,41,42];
(ii) The production of reactive oxygen species (ROS) and reactive nitrogen species (RNS) by MDSC skews the polarization of monocytes, T cells, and macrophages towards anti-inflammatory and regulatory phenotypes [43,44];
(iii) Indirect suppression of T and effector B cells through the induction of tolerogenic immune cells such as de novo generation of Fox-P3^+^ regulatory T cells (Tregs) [45], regulatory B cells, and tumor-associated macrophages (TAMs) [41,46]. Recently, a novel mechanism was elucidated through which MDSC-dependent metabolic and functional paralysis of CD8^+^ T cells occurs [47]. The mechanism involves methylglyoxal-derived glycation of L-arginine products such as argpyrimidine and hydroimidazolone, thereby depleting cytosolic amino acids such as L-arginine, resulting in T-cell paralysis [47,48]. The transfer of methylglyoxal from MDSC to T cells is dependent on cell–cell contact, resulting in more T-cell suppression at sites where MDSC may accumulate, such as tumor tissue [47];
(iv) Secretion of suppressive cytokines such as TGF-β and IL-10 that exert direct suppressive effects on T-cell responses [45];
(v) Induction of T-cell apoptosis through the induction of the B7 family of immune-regulatory ligands, a co-signaling network superfamily that plays an essential role in the modification of T-cell activation and tolerance [49], such as B7-H1 (programmed cell death ligand 1 (PD-L1)), B7-H3, and B7-H4 [50], and impairment of T-cell migration through the reduction of CD62L expression [29].

For the past ~10 years, most immunosuppressive mechanisms have been elucidated from cellular interactions within the tumor microenvironment (TME), driving inferences for MDSC immunosuppressive functions in other conditions.

## 3. MDSC during M. tb Infection

Although research into MDSC and their subtypes originated in cancer biology [51], MDSC are emerging as an important role player during many chronic infectious conditions, most notably in active TB disease [17,18]. MDSC frequencies are heightened in the blood and sputum of patients with pulmonary TB, in healthy persons with acute household exposure to TB (within 3 months of the index case being diagnosed), and in the pleural fluid of patients with extra pulmonary TB, but are reduced to levels similarly seen in healthy controls upon successful anti-TB therapy [29]. In agreement, Knaul et al. showed that MDSC are induced, accumulate, and provide a niche for mycobacterial propagation in a murine TB model, while they regulate lung immune responses and act as M. tb host cells [52]. Other studies have corroborated these findings [53], showing, in addition to elevated frequencies in the periphery, that MDSC frequencies are elevated at the site of infection of patients with active TB disease [54]. Evidence suggests that MDSC play a detrimental role during M. tb infection, inhibiting successful anti-TB immune responses. These effects include the inhibition of T-cell activation, proliferation, trafficking, cytokine responses, and the induction of Tregs [55,56,57,58]. MDSC may also impede the phagocytic responses at the site of disease through the increased production of IL-10 and TGF-b, thereby inhibiting dendritic cell and macrophage function by polarizing them towards a Th2 phenotypic response, as with the responses seen in tumor biology [52]. These impairments are likely to exacerbate disease conditions, impairing the initiation and maintenance of an effective adaptive immune response.

M. tb pathogenesis involves the initial infection of alveolar macrophages (AM), leading to recruitment of other inflammatory myeloid cells and subsequent formation of the classical granuloma [15,16]. As a heterogeneous structure, the granuloma is highly sensitive to oxygen and nutrient gradients that are easily manipulated by the pathogen [16,59]. MDSC have previously been shown to be present in the necrotic areas of the TB granuloma, whereas some report these suppressive myeloid cells at the core or cuff of human and NHP TB granulomas [52,55,60,61,62]. As a result, MDSC compartmentalization in the granuloma is uncertain; however, the knowledge that MDSC can contain the bacteria raises the possibility that MDSC could serve as reservoir cells for M. tb. Knowing this, the metabolic profile of M. tb, MDSC, and the changes MDSC undergo specifically during M. tb infection may provide crucial functional metabolic targets for successful therapeutic interventions.

### M. tb Infection and Metabolic Reprogramming of Myeloid Cells

The metabolic needs of an infected or activated immune cell are significantly different than a resting or non-infected cell [56,63]. Cells use three main pathways to produce energy in the form of ATP and intermediates to sustain cell proliferation, function, and survival: (i) glycolysis, (ii) the Krebs cycle, and (iii) oxidative phosphorylation (OXPHOS) [57]. These are complemented by other pertinent metabolic pathways, namely, (i) the pentose phosphate pathway (PPP), (ii) glutaminolysis, (iii) fatty acid synthesis (FAS), and (iv) fatty acid oxidation (FAO) [58]. Metabolic reprogramming is the process by which cells alter their metabolism to support the energy requirements of their current environment, and immune cells can initiate this through signaling pathways elicited in response to host–pathogen interactions with pattern recognition receptors (PRR), cytokine receptors [64], and host-derived transcriptional regulators such as hypoxia-inducible factor 1α (HIF-1α), mammalian target of rapamycin (mTOR), and glycogen synthase kinase 3 (GSK3) [64,65,66]. The function and response of myeloid cells to infection is shown to be closely tied to their metabolic reprogramming [67,68].

As mentioned above, M. tb pathogenesis involves the initial infection of AM acquired through the inhalation of virulent bacilli. In addition to the AM, other innate immune cells also come in to contact upon inhalation, namely, neutrophils and dendritic cells, which recruit a plethora of other innate cells via the release of pro-inflammatory cytokines such as TNF-α, IL-6, IL-1α, and IL-1β [69,70,71]. Mayito et al. showed that M. tb is able to survive and thrive in myeloid cellular niches such as lipid-rich foamy macrophages [72,73], neutrophils [74,75], dendritic cells, and MDSC [52], as well as in non-myeloid cells such as lymphatic endothelial cells, hemopoietic stem cells, fibroblasts, and adipocytes [76,77]. It is clear that the balance of M. tb infection and interaction with myeloid cells can influence the prognosis of infection [78], and the pathogenicity of M. tb may thus be dependent on its ability to modulate host metabolism [79,80]. Below, we discuss these cells’ metabolisms in relation to M. tb infection.

Immunometabolism of alveolar macrophages: The activation of macrophages necessitates a shift in energy metabolism to meet the needs of the cell as it phagocytoses the offending pathogen [81]. When normal cells transform into cancerous cells, they become highly metabolic and reorganize major metabolic pathways to sustain the rapid pathological proliferation, described as metabolic re-programming [82]. Moreira et al. attributed this increase in glycolysis in tumors to changes in the energetics and redox reactions of cancer cells when compared to normal cells during the cell cycle [83]. The significance of this metabolic polarization is to generate energy under hypoxic environments, especially utilizing carbon for synthesizing biomolecules to sustain rapid tumor growth [84]. Similar mechanisms are observed by macrophages in response to M. tb infection by their polarization from their naïve state (M0) to their pro-inflammatory state (M1). This is accompanied by a metabolic switch from mitochondrial oxidative phosphorylation to hypoxia-inducible factor 1 alpha (HIF-α)-mediated aerobic glycolysis—a phenomenon known as the Warburg effect [85,86]. This metabolic reprogramming was observed in AM during M. tb infection in a murine model of low-dose aerosol infection with early-stage granuloma formation [87]. Host genes including GLUT1, -3, and -6 and glycolytic enzymes/isozymes such as (i) hexokinases (HK) 2 and 3, (ii) phosphofructokinase (PFK) family 1 and 2, (iii) glyceraldehyde-3-phosphate dehydrogenase (GAPDH), (iv) phosphoglycerate kinase 1 (PGK1), (v) enolase 1 (ENO1), (vi) lactate dehydrogenase A (LDHA), and (vii) monocarboxylate transporter 4 (MCT4) were upregulated, reminiscent of the Warburg effect in cancer cells [87].

Although M2 polarization mainly uses mitochondrial oxidative phosphorylation and glutamine metabolism as a major source of carbon and nitrogen, as is usually seen in quiescent or non-polarized macrophages [85,88,89], M. tb has been shown to downregulate enzymes involved in OXPHOS [87]. During the initial stages of infection, a reduction in OXPHOS leads to the production of reactive oxygen species (ROS) and reactive nitrogen species (RNS). The RNS also inhibits the mitochondrial function and electron transport chain (ETC), resulting in reduced redox and increased ROS production [90]. This mechanism has been attributed as one of the ways M. tb perpetuates necrosis of macrophages and facilitates bacterial replication [91].

In vitro studies have shown a switch from OXPHOS to aerobic glycolysis in macrophages following avirulent H37Ra M. tb infection [92]. Conversely, Cumming et al. showed that virulent H37Rv M. tb uniquely decelerates glycolysis and OXPHOS to induce a state of metabolic quiescence and ultimately decrease the rate of ATP production of the macrophage [93]. The authors went on to show that M. tb infection reduced mitochondrial dependency on glucose, increased mitochondrial dependency on fatty acids, and shifted mitochondrial dependency from endogenous fatty acid in uninfected macrophages to exogenous fatty acids for the proliferation of M. tb under stress conditions [93]. This evidence suggests that different lineages of M. tb strains may induce a distinct metabolic reprograming, especially for macrophages [80]. Recently, hydrogen sulfide (H_2_S), a gasotransmitter molecule that plays a key role in several pathologies [94,95], was shown to play a role in M. tb pathogenesis [96]. Researchers have demonstrated that cystathionine γ-lyase (CSE)-deficient mice survive longer than wild-type (WT) mice and sustain decreased pathology and lower bacterial burdens in other organs such as the lung, spleen, and liver [96]. This is accompanied by suppressed central carbon catabolism, specifically the glycolysis and pentose phosphate pathways (PPP)—i.e., depressed glycolysis reduces the secretion of IL-1β and HIF-1α, which has a correlation with the growth of M. tb [96]. These results reveal that fundamental metabolic mechanisms such as glycolysis and endogenous fatty acid metabolism, including establishing the roles of overlooked gaseous signaling molecules such as H_2_S, could be potential candidates for the development of novel drugs that target these energy pathways in M. tb infection [93].

Immunometabolism of neutrophils: These cells participate in the innate immune response observed in early and advanced stages of pulmonary TB infection [97,98] as phagocytes recruited from the pulmonary vasculature to the pulmonary interstitium [99]. Neutrophils eradicate foreign microbes by phagocytosis, the production of ROS, the release of proteolytic enzymes from granules [100], and NETosis (apoptosis caused by the release of condensed chromatin with antimicrobial molecules) [101,102]. The PPP plays a pivotal role in NET release and the diversion of glucose to produce nicotinamide adenine dinucleotide phosphate (NADPH), which feeds NADPH oxidase to produce superoxides. Neutrophils have been shown to rely greatly on glycolysis, especially for inflammatory and phagocytotic functions [103], and inhibitors of OXPHOS do not have any effect on rates of oxygen consumption or hydrogen peroxide (H_2_O_2_) production [104]. On the one hand, inhibition with 2-deoxyglucose (2-DG) (a glucose analogue that can be absorbed by glucose transports (GLUT) but cannot be utilized in glycolysis), severely perturbs the ability of neutrophils to phagocytose and destroy engulfed microbes [105], while the addition of glutamine enhances the phagocytic and killing capacity of in vitro neutrophils [105,106]. Rapid utilization of glycolysis (Warburg effect) seems to be the only way neutrophils synthesize NADPH through the PPP to support their increased respiratory burst, and therefore biochemical pathways of glycolysis and glutaminolysis provide a reliable mechanism for generating reducing equivalents of NADPH used in the microbicidal NADPH oxidase (NOX) system [57].

## 4. Immunometabolism of MDSC in Oncology

Metabolic dysregulation is a hallmark of numerous cancer types [107]. Altered metabolism has been observed in aggressive tumors such as glioblastoma (GBM) and ovarian and gastric cancer. MDSC have been shown to sense or exhibit plasticity in their environment and respond by selecting the most efficient metabolic pathways to sustain their suppressive and pro-tumorigenic functions [108]. In the TME, MDSC increase uptake of FA and activate the switch from glycolysis to FAO [109]. Below, we discuss some of the metabolic adaptations utilized by MDSC:

### 4.1. Metabolic Reprogramming of MDSC

As previously noted, MDSC exhibit distinct functions and phenotypes in various disease settings [42]. They display a certain degree of plasticity where they can assume a pro- or anti-inflammatory phenotype to support tumor proliferation [110]. Although the underlying mechanism leading to MDSC function and activation in TB is not fully known, metabolic reprogramming of MDSC underpins many of the suppressive functions in cancer [42]. As with cancer cells, available evidence indicates that MDSC can undergo anaerobic glycolysis and OXPHOS, which are influenced by substrate availability and by signaling pathways elicited by metabolites or pathogen-derived inflammatory signals [42,111]. During maturation, MDSC exhibit elevated utilization of central carbon metabolism, the PPP, glycolysis, and the TCA cycle [112]. Various signaling pathways, such as the phosphatidylinositol 3-kinase (PI3K)–serine threonine protein kinase (AKT)–mTOR pathway, operate in concert to control metabolic reprogramming/activity in immune cells [113]. Under conditions of oxygen deprivation, mTOR signaling stimulates the HIF-1α pathway, resulting in upregulation of glycolytic enzymes, glucose, and lactate transporters—all of which result in the Warburg effect [47]. The phenotypic heterogeneity of MDSC could cause MDSC to compete with other immune cells for carbon acquisition in the TME and fall under the control of energy metabolic pathways, such as FA metabolism [114], which has a direct effect on the regulation of OXPHOS and glycolysis [114]. Furthermore, the TME may dysregulate some key genes associated with hallmark cancer pathways [115,116], such as HIF-1α (de-regulating cellular energetics), telomerase activation (enabling replicative immortality) [116], and activation of the NF-κβ and TGF-β pathways (antagonizing growth suppressor activity of p53 pathways) [117]. The pulmonary granuloma from M. tb infection may possibly dysregulate some key genes in a similar manner as seen in cancer. HIF-1α binds to the promoter of lactate dehydrogenase A (LDHA), which catalyzes the conversion of pyruvate to lactate, and in some cancers, isoforms of LDHA subunits have the highest efficiency of converting pyruvate to lactate and are linked to increased HIF-1α and VEGF expression, increased tumor size, enhanced metastatic potential, and a poor prognosis [22,118,119]. Non-host immune response therapies such as radiation therapy have also been associated with activation of MDSC and were dependent on enhanced lactate secretion and mediated by HIF-1α activity, resulting in the lactate-regulating MDSC function to reprogram the TME into a more immunosuppressive phenotype [120]. LDH has been found in active pulmonary TB patients’ bronchoalveolar lavage (BAL) fluid at high levels, and BAL LDH corresponds with increased serum LDH (Emad and Rezaian, 1999). Sputum-positive TB patients had higher serum levels of LDH1, LDH2, and LDH3, which have four, three, or two B subunits, respectively [121]. Increases in cerebrospinal fluid (CSF) lactate has also been linked to increased severity of tuberculous meningitis clinical stage [122]. This suggests that LDH levels and isoform specificity, as well as lactate levels, might be used as diagnostic or prognostic markers for pulmonary tuberculosis.

### 4.2. Metabolism of Glucose, Lipids, and Amino Acids by Tumor-Derived MDSC

Hossain et al. found that tumor-infiltrating MDSC (T-MDSC) increased fatty acid uptake and activated FAO, including upregulation of key FAO enzymes [5,123]. These findings exposed new avenues as targets for the development of host-directed therapies to enhance treatment outcomes; inhibiting MDSC has also been shown to improve prospects for successful immunotherapy and/or radiation and chemotherapy [21,124]. Additionally, other metabolic pathways could impact the reconfiguration of host immune responsiveness, such as glycolysis, pentose phosphate pathways (PPP), tricarboxylic cycle (TCA), fatty acid synthesis (FAS), and amino acid synthesis (AAS) [125,126]. T-MDSC have been shown to metabolically shift from glycolysis to OXPHOS and upregulate lipid-associated markers such as CD36 and Msr1 [127]. Studies by Al-Khami et al. showed that the intracellular accumulation of lipids increases the oxidative metabolism and activates the immunosuppressive mechanisms. Inhibition of STAT3 or STAT5 signaling or genetic depletion of the fatty acid translocase CD36 inhibits the activation of oxidative metabolism and the induction of immunosuppressive function in tumor-infiltrating MDSC and results in a CD8^+^ T-cell-dependent delay in tumor growth [127,128]. The TME is characterized by a complex network of blood vessels, tumor cells, and host immune cells, featuring extensive crosstalk of chemokines, cytokines, immune regulatory molecules, and transcription factors that shape the phenotype of MDSC and cancer cells [129,130]. The TME favors a hypoxic environment with a low pH that is thought to arise from the extracellular accumulation of lactate [131] as a result of the interaction of HIF-1α and MYC proto-oncogene transcription factor (Myc) during the upregulation of glycolytic enzymes such as GLUT and the influx of glucose and lactate production [132]. MDSC in tumors have been shown to utilize both glycolysis and OXPHOS, as observed in nasopharyngeal cancer (NPC) [133]. Liu et al. demonstrated that glycolytic activation via the mTOR-pathway was essential for M-MDSC differentiation into M1 and M2 macrophage phenotypes and was dependent on SIRT1 (a negative regulator of mTOR) [134,135]. Furthermore, Cai et al. found that latent membrane protein 1 (LMP1) expression is correlated to multiple glycolytic gene elevation including GLUT1 and production of IL-1β, IL-6, and GM-CSF, resulting in induction of MDSC [133]. Owing to the severely hypoxic nature and heterogenous population of cells within the TME [136], it is no surprise that HIF-1α, which is downstream of the PI3K-AKT-mTOR pathway, is activated in this environment, resulting in the high expression of glucose and glycolytic enzymes with reduced mitochondrial oxygen utilization, in turn facilitating the switch from OXPHOS to glycolysis [137,138]. As a consequence, studies have shown that the upregulation of HIF-1α enhances the suppressive function of MDSC in tumors [138], and even glycolysis by-products such as lactate increase the percentage of MDSC within the TME and solidify their suppressive nature through the HIF-1α pathway [139].

Many similarities exist between TME and TB granulomas. Mature TB granulomas are heterogeneous, and several distinct TB lesions can coexist within the same patient [140]. Histological restationing of the lung tissues from pulmonary TB, sarcoidosis, and lung adenocarcinoma (LUAD) patients showed that TB-infected lung tissue shares a set of potential pathogenic mediators with LUAD. MDSC are considered a major cellular contributor of the suppressive TME [141,142], and tumor-associated growth factors such as granulocyte–macrophage colony-stimulating factor (GM-CSF), granulocyte colony-stimulating factor (G-CSF), and signaling pathways such as STAT3 and STAT5 are known to upregulate lipid transport receptors (such as CD36 and Msr1) in MDSC and enhance lipid accumulation [123]. Similarly, the TB granuloma microenvironment is known to be enriched with lipid content, fatty acids (FAs), cholesterol, and “foamy” macrophages [143,144]. Considering the numerous immune and physical parallels that exist between the TME in cancer and the TB granuloma microenvironment, a similar outcome is anticipated for MDSC in tuberculous granulomas.

Additional support comes from studies demonstrating M2 macrophage dominance during M. tb infection. These rely heavily on OXPHOS for ATP production. Once monocytes enter the interstitial tissue, they differentiate into macrophages and align to an M2 phenotype, although reports suggest that M. tb can induce aerobic glycolysis upon infection [92]. On the contrary, other researchers have shown that M. tb inhibits aerobic glycolysis in infected macrophages [93,145]. Foamy macrophages in TB granulomas have been suggested to participate in sustaining persistent bacteria and tissue pathology that leads to cavitation and the release of infectious bacilli [144]; this may be true for MDSC, which may internalize M. tb, allowing it to flourish in the lipid-rich niche of the granuloma [146,147]. This was demonstrated in cancer, whereby MDSC overloaded with lipids suppressed CD8^+^ T cells, whereas MDSC with normal lipid content did not [147]. In addition, research by Cao et al. showed that fatty acid transport protein 4 (FATP4) is highly expressed in murine tumor-derived MDSC [147]. Other lipids found in MDSC from tumor-bearing mice were found to be in the oxidized form and could be attributed to ROS and myeloperoxidase (MPO) [147,148]. Everything considered, MDSC rely on FAO as a vital fuel source to produce inhibitory cytokines; therefore, targeting this pathway may be a key element in curbing the immunosuppressive nature of MDSC in M. tb infection.

### 4.3. ROS regulates MDSC-Mediated Immune Suppression

**Figure 1:**
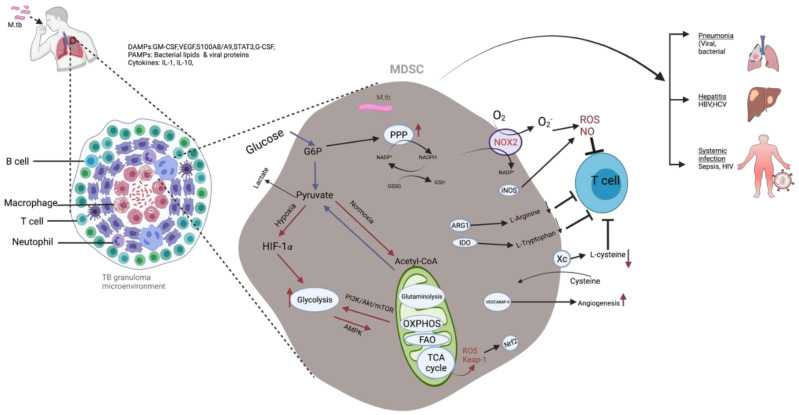
Illustration of how activated MDSC maintain redox homeostasis and how energy metabolism contributes to the immunosuppressive role of MDSC. Once MDSC are activated there is an increase in carbon metabolism in pathways such as glycolysis, PPP, and the TCA cycle, as represented by the red arrows [150]. Upregulation of Nrf2 results in increased expression of antioxidant genes such as Keap-1 and metabolic reprogramming of MDSC [160,161]. Glutathione (GSH) is produced as a result of PPP elevation and is pivotal as an antioxidant and for differentiation of MDSC [162]. MDSC promote M. tb intracellular replication, and bacilli can be found in the granuloma’s central necrotic region, while T cells, B cells, Tregs, and MDSC also migrate to the granuloma. A combination of cytokines and direct stimulation of specific microbial receptors by various microorganisms may be used to activate or reprogram circulating immune cells and MDSC. MDSC are recruited in various organs, where they suppress the disease and modulate its manifestations and outcome. Cognizance should be made that MDSC counteract the effect of ROS derived from OXPHOS by utilization and upregulation of glycolytic genes and glycolysis [155]. Figure created with BioRender.com.

Signals such as ROS and NO have been well researched and documented to form signatures of human immune responses in oncology [149]. ROS are by-products of cellular oxidative metabolism [149]; in a healthy organism they help sustain an internal redox environment that balances free radicals produced by cellular antioxidants and enzyme systems [149], but during cancer they are produced by MDSC as a mechanism of suppressive function [150,151]. Studies by Nagaraj et al. highlighted that ROS and peroxynitrite production by MDSC has the potential to modify CD8^+^ T cells so that they lose their capacity to bind major histocompatibility complex (MHC) molecules and induce antigen-specific tolerance of peripheral CD8^+^ T cells [152]. By-products such as H_2_O_2_ formed from MDSC interaction with superoxide attenuate T-cell CD3ζ expression and consequently inactivate T cells and reduce IFN-γ expression [150]. In the lung, the initial encounter of alveolar macrophages and M. tb bacilli induces an oxidative burst [152,153] leading to the production of ROS that offers resistance to the growth of bacteria or other invading microorganisms [153,154]. Energy-generating pathways are a critical source of ROS, and Jian et al. showed that MDSC counteract OXPHOS via upregulation of glycolysis. They also identified the glycolytic metabolite, phosphoenolpyruvate (PEP), as a pivotal antioxidant that prevents excess ROS production by MDSC, resulting in protection from apoptosis [155] (Figure 1). Other signaling molecules such as HIF-1α and nuclear respiratory factor (NRF) are critical in conditions of hypoxia and pathways that modulate MDSC differentiation, allowing them to take a dual M1/M2 phenotype and mTOR-induced glycolytic activities [32,134,156] and contribute to immune cell reprogramming. In addition, Nrf2 promotes the expression of NAD(P)H-quinone oxidoreductase 1 (NQO1), the enzyme responsible for the reduction and detoxification of reactive quinones, and the enzyme responsible for the cleavage of heme into biliverdin, heme oxygenase of expression-1 (HMOX1) [157]. Interestingly, HMOX1 functions in part as an antioxidant but has also been shown to be anti-inflammatory, reducing IL-12 p40, IL-16, and TNF-α levels in dendritic cells and in allergic airways [158]. In contrast to HIF-1α, AMP-activated protein kinase (AMPK) exerts immunosuppressive function by inhibiting glycolysis through the PI3K–AKT–mTOR pathway. AMPK drives glycolysis toward OXPHOS during glucose metabolism.

It is clear from the literature that MDSC thrive in high-ROS environments and upregulate ROS production, which is one of the keyways they confer immuno-regulatory effects on immune cells [150,151,159]. It is possible, considering the similarities between the TME and the TB granuloma microenvironments, that the same mechanism may be present/active in the TB granuloma, where MDSC can accumulate and thrive in an ROS environment and enhance the suppressive effect of MDSC.

## 5. An Intersection Point of MDSC in Tumor Biology and MDSC in Tuberculosis

Chronic inflammation and fibrosis are two major characteristics of TB infection, and these factors have been linked to the induction of genetic mutations and alterations in lung parenchyma tissue, which is involved in both diseases [163]. The overlap of immune mediators such as interleukins and the discovery of increased frequencies of MDSC in both TB [29] and cancer [164] all contribute to the hypothesis that tumor-derived MDSC functions translate eloquently to MDSC functions in TB [163,165]. Because of their suppressive role in the TME, contributing to metabolite production, depletion of oxygen levels, and metabolite production [47,166], MDSC have become a target for many host-directed therapies (HDTs) to limit MDSC in TB [167]. Although essential nutrient levels may decrease in the TME compared to the normal tissue niche, several metabolic entities such as lactate, glutamate, or free fatty acids increase, which may be responsible for modulating cancer progression and the corresponding immune response [65]. The TME is populated by both cancerous and non-cancerous cells, and the influence of metabolite content on each cell type is interconnected, making it critical to understand metabolic crosstalk within these cells to identify novel therapeutic targets. Using these insights from tumor biology to develop therapeutics that target MDSC in M. tb infection would be ideal for adjunct treatment during standard tuberculosis treatment.

### 5.1. Crosstalk between MDSC and B Cells

**Figure 2:**
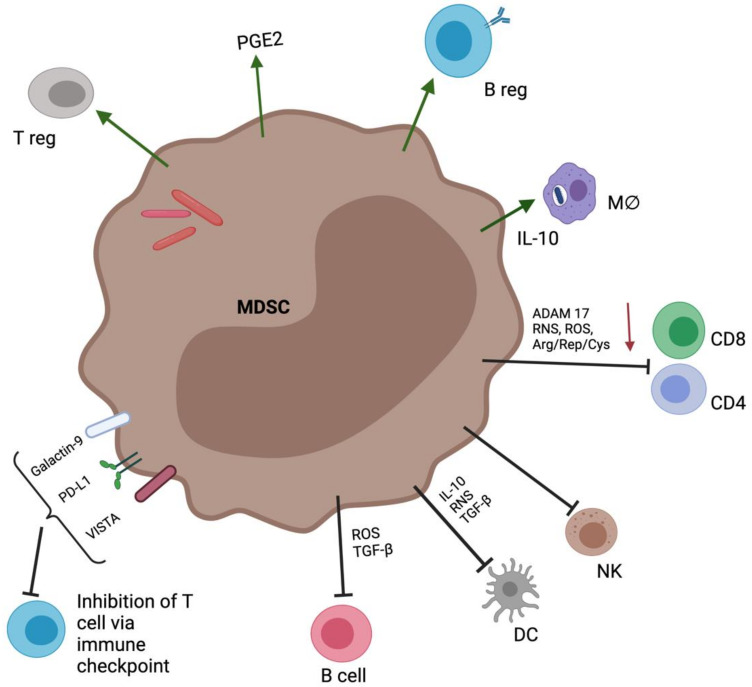
The crosstalk between MDSC and immune cells. MDSC suppress T-cell function and directly result in inhibition or loss of function. Color-coded arrows indicate induction or activation (green) or suppression (black and red). Immune suppression by MDSC is mainly antigen specific and contact dependent and utilizes several major pathways (not shown here). Elimination of key nutrition factors for T cells from the microenvironment (e.g., L-arginine). Disruption of homing and trafficking of T cells (through the expression of ADAM17, the nitration of CCL2). Upregulation of immune checkpoint, such as PD-Ll, galectin-9, and VISTA. M-MDSC restrict proliferation and release of cytokines by effector CD4 and CD8 lymphocytes and induce apoptotic cell death. Abbreviations: ADAM 17, ADAM metallopeptidase domain 17; ARG1, arginase 1; CD, cluster of differentiation; DC, dendritic cell; IDO1, indoleamine dioxygenase 1; IFN-γ, interferon gamma; IL-10, interleukin 10; l-Arg, l-arginine; l-Cys, l-cysteine; MΦ, macrophage; NK, natural killer cell; NKGD2, killer cell lectin like receptor K1; PGE2, prostaglandin E2; PD-L1, programmed-death ligand 1; RNS, reactive nitrogen species; ROS, reactive oxygen species; TGF-β, transforming growth factor beta; Trp, tryptophan; VISTA, V-domain Ig suppressor of T-cell activation.

Numerous studies have demonstrated that MDSC-mediated immunosuppression has the potential to inhibit innate and adaptive immune cell activation, proliferation, viability, trafficking, and cytokine production. MDSC use a variety of suppressive mechanisms, and their ability to initiate antigen-specific versus non-specific suppression is likely to differ [154,168]. MDSC interaction with myeloid cells such as DC, neutrophils, and macrophages is also limited, with most findings demonstrating that their inhibitory effects are amplified by cross-regulation with macrophages at tumor sites. In lung infections such as *Pneumocystis pneumonia* (PcP), M-MDSC-expressing PD-L1 are activated, impairing phagocytic activity while increasing PD-1 expression in AM [32,155]. MDSC have been shown in mice infected with *K. pneumoniae* or challenged with LPS to efferocytose-infected apoptotic neutrophils [156]. A thorough and more detailed discussion of MDSC immunosuppression and cellular interactions is reviewed by Dorhoi and Du Plessis [32], whereas MDSC involvement in lipid raft generation during M. tb infection and the cellular interactions required is reviewed elsewhere [75]. Figure 2 depicts some of the cellular interactions with MDSC.

In a study of murine infected with the lymphocytic choriomeningitis virus (LCMV), virus-specific B cells interacting with myeloid cells were inhibited and eventually killed by a population of CD11b^+^Ly6C^hi^ inflammatory monocytes [157]. These myeloid cells were recruited through a type-1 interferon (IFN-1) and CCR2-dependent mechanism, and they suppressed antiviral B-cell response by virtue of their ability to produce NO [157]. Similarly, Fooksman et al. also showed that macrophages and Gr-1-negative monocytes such as the CX3CR1^+^ subset were able to dampen the numbers of antibody-secreting cells (ASC) and titers possibly through secretion of IL-10. They also observed that IL-6 enhanced ASC production, although L-6 was not required by myeloid cells to dampen ASC in the lymph node [158]. Although these monocytic cells phenotypically resemble M-MDSC, the role of MDSC in regulating B cells has been understudied. Recently it was found that PMN-MDSC differentially regulate B-cell function, especially during proliferation and antibody production, which is stimulant dependent [159]. They also showed that MDSC-mediated effects are cell contact-dependent and included NO, arginase-1, ROS, and apoptosis [159], including the reduction of second signal cell surface molecules of APCs CD80 (B7-1) and CD86 (B7-2). The involvement of cell–cell contact could suggest that MDSC act either through cell surface receptors or through the release of soluble mediators that are short-lived [38], as demonstrated in Figure 2. M-MDSC from murine leukemia LP-BM5 retrovirus-infected mice were also shown to suppress B-cell responsiveness in a contact-independent manner and lowered the levels of IL-10 released by regulatory B (Breg) cells in response to LPS stimulation. This process was dependent on mediators such as superoxide, peroxynitrite, nitric oxide and TGF-β [160]. Furthermore, a mouse model of systemic lupus erythematosus (SLE) concluded that MDSC induced the expansion of Breg cells through iNOS and ceased autoimmune activity [161].

Resting naïve recirculating B cells have been shown to accumulate GSK3, a metabolic sensor that incorporates cytokine-induced proliferation with nutrient availability and is known to inhibit B-cell growth and inhibit metabolic activity and proliferation under hypoxic conditions [65]. Using a mouse collagen-induced arthritis (CIA) model, Cook et al. demonstrated that CD40L/IL-4-stimulated B cells are inhibited by autologous M-MDSC. This interaction inhibited antibody secretion in vitro and reduced serum levels of antigen-specific antibodies after adoptive transfer in autoimmune disease [162]. Thus, it would be of interest to determine whether MDSC can inhibit B-cell antibody activity in M. tb infection and aid instead the accumulation of Breg cells.

### 5.2. The Impact of MDSC in TB Infection and Prospects of Immune Metabolic Targeting Therapy of Drug-Resistant TB

The recognition that MDSC could play a pivotal role in the chronicity of infection and may contribute to treatment failure of anti-TB medication warrants further research. It is likely that the strategies shown to be useful in cancer immunotherapy may also be useful for other pathologies where elimination or inhibition of MDSC is a therapeutic aim. An ideal drug or compound would be one that can induce cellular selectivity and take advantage of the differential metabolic requirements of effector and regulatory immune cells during an immune response [163]. Given that mycobacterial metabolism is linked to host cellular metabolism and that M. tb can metabolize various host metabolites (including lactate, pyruvate, and cholesterol) as a nutrient source, targeting metabolic pathways therapeutically should specifically target cells of interest such as MDSC, with limited effects on other immune cells, hence reducing the side effects and improving prognosis. Multiple compounds are undergoing research for immunomodulation in cancer, and known drugs are being repurposed in mice and humans as immunotherapy, whereas others have been tested in both pre-clinical and clinical settings [164]. These either inhibit the recruitment and expansion of MDSC or eliminate the MDSC population. Metformin is a widely used antidiabetic drug that inhibits the frequency and recruitment of MDSC in cancer by modulating the expression and activity of HIF-α and other targets [165,166]. Metformin was also shown to reduce disease severity and inflammation in diabetic patients who happened to take metformin during TB treatment [167].

Understanding how host cells and MDSC metabolism can influence the outcome of M. tb infection is cardinal. The rise of drug-resistant mutations in M. tb strains seem to mediate changes in cell-wall biosynthesis and bacterial metabolism, which suggests that drug-resistant M. tb modulates changes in cell-wall biosynthesis and bacterial metabolism. Therefore, future studies are needed to closely look at the relationship of MDSC metabolism during infection with various clinical strains.

## 6. Conclusions

Evidence strongly suggests that manipulation of cellular metabolism ultimately directs the phenotype and physiological function of immune cells to either a pro-inflammatory or anti-inflammatory phenotype. There is, however, no universal agreement on the metabolic preference of MDSC, and it is likely that MDSC subsets may utilize specific metabolic pathways during certain infection stages that could be distinct from those used by other immune cells. The adaptable nature of M-MDSC has been reflected by the utilization FAO in tumors as a source of ATP, whereas PMN-MDSC preferentially engage in glycolysis and OXPHOS [123]. Likewise, a similar scenario in the TB granuloma microenvironment can be envisioned where there is competition for nutrients, and oxygen could force MDSC and other myeloid cells to adapt their metabolism by selecting a pathway that is most sustainable for energy production. The involvement and elevation of MDSC in chronic diseases such as M. tb are ideal targets owing to their suppressive nature, high plasticity, and differentiation potential.

Tumors and late-stage TB granulomas share multiple features, including hypoxia, neovascularization, and chronic inflammation, which shape the cellular landscape of such environments [65]. Research in cancer shows that even lipid metabolism is altered (whereby both subsets of MDSC can undergo metabolic reprogramming and increase fatty acid β oxidation [128]) in MDSC, suggesting that the same might be the case in TB. We speculate that, during distinct stages of M. tb infection, MDSC could be metabolically reprogrammed with pharmacological agents through manipulation of immune cell receptors upregulated mainly on MDSC, such as those linked to distinct energy pathways (e.g., lipid-associated marker CD36 and lipid transport proteins [127], including those expressing LOX1 [168], or by inhibition of FAO, which would result in heightened mitochondrial electron function and TCA. For instance, strategies designed for targeting MDSC in cancer could also be adapted for use in advanced or untreatable TB, as both these conditions demonstrate chronic inflammatory profiles and harbor MDSC. This strategy may also be useful in drug-resistant TB or post-TB lung disease. A confounding factor is that to date, there has been no drug that exclusively targets MDSC, although several cancer clinical trial compounds have shown promise, albeit not for M. tb infection. It will be important to elucidate the differences in MDSC metabolic pathways in the TB granuloma versus those circulating in blood compared to other diseases. Advances in the cancer field strongly encourage research into the metabolic pathways effective in myeloid cells, such as MDSC, during various stages of pulmonary TB to inform the development of targets regulating MDSC metabolism, such as glucose, lipid, and AA metabolism to advance precision therapy in latent, sub-clinical, and active TB.

## References

[1] Emens L.A., Silverstein S.C., Khleif S., Marincola F.M., Galon J. (2012). Toward integrative cancer immunotherapy: Targeting the tumor microenvironment. J. Transl. Med..

[2] Donkor M.K., Lahue E., Hoke T.A., Shafer L.R., Coskun U., Solheim J.C., Gulen D., Bishay J., Talmadge J.E. (2009). Mammary tumor heterogeneity in the expansion of myeloid-derived suppressor cells. Int. Immunopharmacol..

[3] Gabrilovich D.I., Ostrand-Rosenberg S., Bronte V. (2012). Coordinated regulation of myeloid cells by Tumors. Nat. Rev. Immunol..

[4] Umansky V., Blattner C., Gebhardt C., Utikal J. (2016). The role of myeloid-derived suppressor cells (MDSC) in cancer progression. Vaccines.

[5] Hossain F., Al-Khami A.A., Wyczechowska D., Hernandez C., Zheng L., Reiss K., Del Valle L., Trillo-Tinoco J., Maj T., Zou W. (2015). Inhibition of Fatty Acid Oxidation Modulates Immunosuppressive Functions of Myeloid-Derived Suppressor Cells and Enhances Cancer Therapies. Cancer Immunol. Res..

[6] Park M.-J., Baek J.-A., Kim S.-Y., Park S.-H., Cho M.-L., Kwok S.-K. (2019). 268 PD-L1 expressing myeloid-derived suppressor cells (MDSCs) have potent immuoregulatory activity and control lupus-like autoimmunity. Abstracts.

[7] Forghani P., Petersen C.T., Waller E.K. (2017). Activation of VIP signaling enhances immunosuppressive effect of MDSC on CMV-induced adaptive immunity. Oncotarget.

[8] Youn J.I., Gabrilovich D.I. (2010). The biology of myeloid-derived suppressor cells: The blessing and the curse of morphological and functional heterogeneity. Eur. J. Immunol..

[9] Porembka M.R., Mitchem J.B., Belt B.A., Hsieh C.-S., Lee H.-M., Herndon J., Gillanders W.E., Linehan D.C., Goedegebuure P. (2012). Pancreatic adenocarcinoma induces bone marrow mobilization of myeloid-derived suppressor cells which promote primary tumor growth. Cancer Immunol. Immunother..

[10] Wang L., Chang E.W., Wong S.C., Ong S.-M., Chong D.Q., Ling K.L. (2013). Increased myeloid-derived suppressor cells in gastric cancer correlate with cancer stage and plasma S100A8/A9 proinflammatory proteins. J. Immunol..

[11] Diaz-Montero C.M., Salem M.L., Nishimura M.I., Garrett-Mayer E., Cole D.J., Montero A.J. (2009). Increased circulating myeloid-derived suppressor cells correlate with clinical cancer stage, metastatic tumor burden, and doxorubicin–cyclophosphamide chemotherapy. Cancer Immunol. Immunother..

[12] Cao D.Y., Spivia W.R., Veiras L.C., Khan Z., Peng Z., Jones A.E., Bernstein E.A., Saito S., Okwan-Duodu D., Parker S.J. (2020). ACE overexpression in myeloid cells increases oxidative metabolism and cellular ATP. J. Biol. Chem..

[13] Schulte-Schrepping J., Reusch N., Paclik D., Baßler K., Schlickeiser S., Zhang B., Krämer B., Krammer T., Brumhard S., Bonaguro L. (2020). Severe COVID-19 is marked by a dysregulated myeloid cell compartment. Cell.

[14] Cooper A.M., Torrado E. (2012). Protection versus pathology in tuberculosis: Recent insights. Curr. Opin. Immunol..

[15] Qualls J.E., Murray P.J. (2016). Immunometabolism within the tuberculosis granuloma: Amino acids, hypoxia, and cellular respiration. Semin. Immunopathol..

[16] Ost M., Singh A., Peschel A., Mehling R., Rieber N., Hartl D. (2016). Myeloid-derived suppressor cells in bacterial infections. Front. Cell. Infect. Microbiol..

[17] Janols H., Bergenfelz C., Allaoui R., Larsson A.M., Rydén L., Björnsson S., Janciauskiene S., Wullt M., Bredberg A., Leandersson K. (2014). A high frequency of MDSC in sepsis patients, with the granulocytic subtype dominating in gram-positive cases. J. Leukoc. Biol..

[18] Singer M., Deutschman C.S., Seymour C.W., Shankar-Hari M., Annane D., Bauer M., Bellomo R., Bernard G.R., Chiche J.-D., Coopersmith C.M. (2016). The third international consensus definitions for sepsis and septic shock (Sepsis-3). JAMA.

[19] Periasamy S., Avram D., McCabe A., MacNamara K.C., Sellati T.J., Harton J.A. (2016). An Immature Myeloid/Myeloid-Suppressor Cell Response Associated with Necrotizing Inflammation Mediates Lethal Pulmonary Tularemia. PLoS Pathog..

[20] Du Plessis N., Loebenberg L., Kriel M., von Groote-Bidlingmaier F., Ribechini E., Loxton A.G., van Helden P.D., Lutz M.B., Walzl G. (2013). Increased Frequency of Myeloid-derived Suppressor Cells during Active Tuberculosis and after Recent *Mycobacterium tuberculosis* Infection Suppresses T-Cell Function. Am. J. Respir. Crit. Care Med..

[21] Yan D., Adeshakin A.O., Xu M., Afolabi L.O., Zhang G., Chen Y.H., Wan X. (2019). Lipid Metabolic Pathways Confer the Immunosuppressive Function of Myeloid-Derived Suppressor Cells in Tumor. Front. Immunol..

[22] Al-Khami A.A., Rodriguez P.C., Ochoa A.C. (2016). Metabolic reprogramming of myeloid-derived suppressor cells (MDSC) in cancer. OncoImmunology.

[23] Norata G.D., Caligiuri G., Chavakis T., Matarese G., Netea M.G., Nicoletti A., O’Neill L.A., Marelli-Berg F.M. (2015). The cellular and molecular basis of translational immunometabolism. Immunity.

[24] Pearce E.L., Pearce E.J. (2013). Metabolic pathways in immune cell activation and quiescence. Immunity.

[25] Filipazzi P., Huber V., Rivoltini L. (2012). Phenotype, function and clinical implications of myeloid-derived suppressor cells in cancer patients. Cancer Immunol. Immunother..

[26] Condamine T., Ramachandran I., Youn J.-I., Gabrilovich D.I. (2015). Regulation of tumor metastasis by myeloid-derived suppressor cells. Annu. Rev. Med..

[27] Wang W., Xia X., Mao L., Wang S. (2019). The CCAAT/enhancer-binding protein family: Its roles in MDSC expansion and function. Front. Immunol..

[28] Peranzoni E., Zilio S., Marigo I., Dolcetti L., Zanovello P., Mandruzzato S., Bronte V. (2010). Myeloid-derived suppressor cell heterogeneity and subset definition. Curr. Opin. Immunol..

[29] Dumitru C.A., Moses K., Trellakis S., Lang S., Brandau S. (2012). Neutrophils and granulocytic myeloid-derived suppressor cells: Immunophenotyping, cell biology and clinical relevance in human oncology. Cancer Immunol. Immunother..

[30] Solito S., Marigo I., Pinton L., Damuzzo V., Mandruzzato S., Bronte V. (2014). Myeloid-derived suppressor cell heterogeneity in human cancers. Ann. N. Y. Acad. Sci..

[31] Mandruzzato S., Brandau S., Britten C.M., Bronte V., Damuzzo V., Gouttefangeas C., Maurer D., Ottensmeier C., van der Burg S.H., Welters M.J.P. (2016). Toward harmonized phenotyping of human myeloid-derived suppressor cells by flow cytometry: Results from an interim study. Cancer Immunol. Immunother..

[32] Dorhoi A., Du Plessis N. (2017). Monocytic myeloid-derived suppressor cells in chronic infections. Front. Immunol..

[33] Zhao F., Hoechst B., Duffy A., Gamrekelashvili J., Fioravanti S., Manns M.P., Greten T.F., Korangy F. (2012). S100A9 a new marker for monocytic human myeloid-derived suppressor cells. Immunology.

[34] Chai E., Zhang L., Li C. (2019). LOX-1+ PMN-MDSC enhances immune suppression which promotes glioblastoma multiforme progression. Cancer Manag. Res..

[35] Veglia F., Tyurin V.A., Blasi M., De Leo A., Kossenkov A.V., Donthireddy L., To T.K.J., Schug Z., Basu S., Wang F. (2019). Fatty acid transport protein 2 reprograms neutrophils in cancer. Nature.

[36] Bronte V., Brandau S., Chen S.-H., Colombo M.P., Frey A.B., Greten T.F., Mandruzzato S., Murray P.J., Ochoa A., Ostrand-Rosenberg S. (2016). Recommendations for myeloid-derived suppressor cell nomenclature and characterization standards. Nat. Commun..

[37] Goldmann O., Beineke A., Medina E. (2017). Identification of a Novel Subset of Myeloid-Derived Suppressor Cells During Chronic Staphylococcal Infection That Resembles Immature Eosinophils. J. Infect. Dis..

[38] Gabrilovich D.I., Nagaraj S. (2009). Myeloid-derived suppressor cells as regulators of the immune system. Nat. Rev. Immunol..

[39] Parker K.H., Sinha P., Horn L.A., Clements V.K., Yang H., Li J., Tracey K.J., Ostrand-Rosenberg S. (2014). HMGB1 enhances immune suppression by facilitating the differentiation and suppressive activity of myeloid-derived suppressor cells. Cancer Res..

[40] Chiba Y., Mizoguchi I., Hasegawa H., Ohashi M., Orii N., Nagai T., Sugahara M., Miyamoto Y., Xu M., Owaki T. (2018). Regulation of myelopoiesis by proinflammatory cytokines in infectious diseases. Cell. Mol. Life Sci..

[41] Rajabinejad M., Salari F., Gorgin Karaji A., Rezaiemanesh A. (2019). The role of myeloid-derived suppressor cells in the pathogenesis of rheumatoid arthritis; anti-or pro-inflammatory cells?. Artif. Cells Nanomed. Biotechnol..

[42] Li T., Mao C., Wang X., Shi Y., Tao Y. (2020). Epigenetic crosstalk between hypoxia and tumor driven by HIF regulation. J. Exp. Clin. Cancer Res..

[43] Chiu D.K.-C., Tse A.P.-W., Xu I.M.-J., Di Cui J., Lai R.K.-H., Li L.L., Koh H.-Y., Tsang F.H.-C., Wei L.L., Wong C.-M. (2017). Hypoxia inducible factor HIF-1 promotes myeloid-derived suppressor cells accumulation through ENTPD2/CD39L1 in hepatocellular carcinoma. Nat. Commun..

[44] Alfaro C., Teijeira A., Oñate C., Pérez G., Sanmamed M.F., Andueza M.P., Alignani D., Labiano S., Azpilikueta A., Rodriguez-Paulete A. (2016). Tumor-produced interleukin-8 attracts human myeloid-derived suppressor cells and elicits extrusion of neutrophil extracellular traps (NETs). Clin. Cancer Res..

[45] Yao L., Abe M., Kawasaki K., Akbar S.M.F., Matsuura B., Onji M., Hiasa Y. (2016). Characterization of liver monocytic myeloid-derived suppressor cells and their role in a murine model of non-alcoholic fatty liver disease. PLoS ONE.

[46] Condamine T., Gabrilovich D.I. (2011). Molecular mechanisms regulating myeloid-derived suppressor cell differentiation and function. Trends Immunol..

[47] Fleming V., Hu X., Weber R., Nagibin V., Groth C., Altevogt P., Utikal J., Umansky V. (2018). Targeting myeloid-derived suppressor cells to bypass tumor-induced immunosuppression. Front. Immunol..

[48] Rodríguez P.C., Ochoa A.C. (2008). Arginine regulation by myeloid derived suppressor cells and tolerance in cancer: Mechanisms and therapeutic perspectives. Immunol. Rev..

[49] Hu C., Pang B., Lin G., Zhen Y., Yi H. (2019). Energy metabolism manipulates the fate and function of Tumor myeloid-derived suppressor cells. Br. J. Cancer.

[50] Köstlin N., Vogelmann M., Spring B., Schwarz J., Feucht J., Härtel C., Orlikowsky T.W., Poets C.F., Gille C. (2017). Granulocytic myeloid-derived suppressor cells from human cord blood modulate T-helper cell response towards an anti-inflammatory phenotype. Immunology.

[51] Derive M., Bouazza Y., Alauzet C., Gibot S. (2012). Myeloid-derived suppressor cells control microbial sepsis. Intensive Care Med..

[52] Huang B., Pan P.-Y., Li Q., Sato A.I., Levy D.E., Bromberg J., Divino C.M., Chen S.-H. (2006). Gr-1+ CD115+ immature myeloid suppressor cells mediate the development of tumor-induced T regulatory cells and T-cell anergy in tumor-bearing host. Cancer Res..

[53] Wang Y., Schafer C.C., Hough K.P., Tousif S., Duncan S.R., Kearney J.F., Ponnazhagan S., Hsu H.-C., Deshane J.S. (2018). Myeloid-derived suppressor cells impair B cell responses in lung cancer through IL-7 and STAT5. J. Immunol..

[54] Baumann T., Dunkel A., Schmid C., Schmitt S., Hiltensperger M., Lohr K., Laketa V., Donakonda S., Ahting U., Lorenz-Depiereux B. (2020). Regulatory myeloid cells paralyze T cells through cell–cell transfer of the metabolite methylglyoxal. Nat. Immunol..

[55] Tsiganov E.N., Verbina E.M., Radaeva T.V., Sosunov V.V., Kosmiadi G.A., Nikitina I.Y., Lyadova I.V. (2014). Gr-1^dim^CD11b^+^ Immature Myeloid-Derived Suppressor Cells but Not Neutrophils Are Markers of Lethal Tuberculosis Infection in Mice. J. Immunol..

[56] El Daker S., Sacchi A., Tempestilli M., Carducci C., Goletti D., Vanini V., Colizzi V., Lauria F.N., Martini F., Martino A. (2015). Granulocytic myeloid derived suppressor cells expansion during active pulmonary tuberculosis is associated with high nitric oxide plasma level. PLoS ONE.

[57] Lenaerts A., Barry C.E., Dartois V. (2015). Heterogeneity in tuberculosis pathology, microenvironments and therapeutic responses. Immunol. Rev..

[58] Dorhoi A., Kaufmann S.H. (2015). Versatile myeloid cell subsets contribute to tuberculosis-associated inflammation. Eur. J. Immunol..

[59] Rabbani N., Thornalley P.J. (2015). Dicarbonyl stress in cell and tissue dysfunction contributing to ageing and disease. Biochem. Biophys. Res. Commun..

[60] Capece D., Verzella D., Fischietti M., Zazzeroni F., Alesse E. (2012). Targeting costimulatory molecules to improve antitumor immunity. BioMed Res. Int..

[61] Fujimura T., Ring S., Umansky V., Mahnke K., Enk A.H. (2012). Regulatory T cells stimulate B7-H1 expression in myeloid-derived suppressor cells in ret melanomas. J. Investig. Dermatol..

[62] Knaul J.K., Jörg S., Oberbeck-Mueller D., Heinemann E., Scheuermann L., Brinkmann V., Mollenkopf H.-J., Yeremeev V., Kaufmann S.H., Dorhoi A. (2014). Lung-residing myeloid-derived suppressors display dual functionality in murine pulmonary tuberculosis. Am. J. Respir. Crit. Care Med..

[63] Obregón-Henao A., Henao-Tamayo M., Orme I.M., Ordway D.J. (2013). Gr1intCD11b+ myeloid-derived suppressor cells in Mycobacterium tuberculosis infection. PLoS ONE.

[64] Zahorchak A.F., Ezzelarab M.B., Lu L., Turnquist H.R., Thomson A.W. (2016). In Vivo Mobilization and Functional Characterization of Nonhuman Primate Monocytic Myeloid-Derived Suppressor Cells. Am. J. Transpl..

[65] Dorhoi A., Kotzé L.A., Berzofsky J.A., Sui Y., Gabrilovich D.I., Garg A., Hafner R., Khader S.A., Schaible U.E., Kaufmann S.H. (2020). Therapies for tuberculosis and AIDS: Myeloid-derived suppressor cells in focus. J. Clin. Investig..

[66] Eisenreich W., Rudel T., Heesemann J., Goebel W. (2019). How viral and intracellular bacterial pathogens reprogram the metabolism of host cells to allow their intracellular replication. Front. Cell. Infect. Microbiol..

[67] Ganeshan K., Chawla A. (2014). Metabolic regulation of immune responses. Annu. Rev. Immunol..

[68] Stine Z.E., Walton Z.E., Altman B.J., Hsieh A.L., Dang C.V. (2015). MYC, metabolism, and cancer. Cancer Discov..

[69] Cazzaniga M., Bonanni B. (2015). Relationship between metabolic reprogramming and mitochondrial activity in cancer cells. Understanding the anticancer effect of metformin and its clinical implications. Anticancer Res..

[70] Jellusova J., Cato M.H., Apgar J.R., Ramezani-Rad P., Leung C.R., Chen C., Richardson A.D., Conner E.M., Benschop R.J., Woodgett J.R. (2017). Gsk3 is a metabolic checkpoint regulator in B cells. Nat. Immunol..

[71] Escoll P., Buchrieser C. (2018). Metabolic reprogramming of host cells upon bacterial infection: Why shift to a Warburg-like metabolism?. FEBS J..

[72] O’Neill L.A., Kishton R.J., Rathmell J. (2016). A guide to immunometabolism for immunologists. Nat. Rev. Immunol..

[73] Domblides C., Lartigue L., Faustin B. (2018). Metabolic stress in the immune function of T cells, macrophages and dendritic cells. Cells.

[74] Cohen S.B., Gern B.H., Delahaye J.L., Adams K.N., Plumlee C.R., Winkler J.K., Sherman D.R., Gerner M.Y., Urdahl K.B. (2018). Alveolar macrophages provide an early *Mycobacterium tuberculosis* niche and initiate dissemination. Cell Host Microbe.

[75] Kotzé L.A., Young C., Leukes V.N., John V., Fang Z., Walzl G., Lutz M.B., du Plessis N. (2020). *Mycobacterium tuberculosis* and myeloid-derived suppressor cells: Insights into caveolin rich lipid rafts. EBioMedicine.

[76] Law K., Weiden M., Harkin T., Tchou-Wong K., Chi C., Rom W.N. (1996). Increased release of interleukin-1 beta, interleukin-6, and tumor necrosis factor-alpha by bronchoalveolar cells lavaged from involved sites in pulmonary tuberculosis. Am. J. Respir. Crit. Care Med..

[77] Kim M.J., Wainwright H.C., Locketz M., Bekker L.G., Walther G.B., Dittrich C., Visser A., Wang W., Hsu F.F., Wiehart U. (2010). Caseation of human tuberculosis granulomas correlates with elevated host lipid metabolism. EMBO Mol. Med..

[78] Russell D.G., VanderVen B.C., Lee W., Abramovitch R.B., Kim M.-j., Homolka S., Niemann S., Rohde K.H. (2010). *Mycobacterium tuberculosis* wears what it eats. Cell Host Microbe.

[79] Mayito J., Andia I., Belay M., Jolliffe D.A., Kateete D.P., Reece S.T., Martineau A.R. (2018). Anatomic and Cellular Niches for *Mycobacterium tuberculosis* in Latent Tuberculosis Infection. J. Infect. Dis..

[80] Behr M.A., Waters W.R. (2014). Is tuberculosis a lymphatic disease with a pulmonary portal?. Lancet Infect. Dis..

[81] Mariotti S., Sargentini V., Pardini M., Giannoni F., De Spirito M., Gagliardi M.C., Greco E., Teloni R., Fraziano M., Nisini R. (2013). *Mycobacterium tuberculosis* may escape helper T cell recognition by infecting human fibroblasts. Hum. Immunol..

[82] Neyrolles O., Hernández-Pando R., Pietri-Rouxel F., Fornès P., Tailleux L., Payán J.A.B., Pivert E., Bordat Y., Aguilar D., Prévost M.-C. (2006). Is adipose tissue a place for *Mycobacterium tuberculosis* persistence?. PLoS ONE.

[83] Davis J.M., Ramakrishnan L. (2009). The Role of the Granuloma in Expansion and Dissemination of Early Tuberculous Infection. Cell.

[84] Bénard A., Sakwa I., Schierloh P., Colom A., Mercier I., Tailleux L., Jouneau L., Boudinot P., Al-Saati T., Lang R. (2018). B cells producing type I IFN modulate macrophage polarization in tuberculosis. Am. J. Respir. Crit. Care Med..

[85] Howard N.C., Marin N.D., Ahmed M., Rosa B.A., Martin J., Bambouskova M., Sergushichev A., Loginicheva E., Kurepina N., Rangel-Moreno J. (2018). *Mycobacterium tuberculosis* carrying a rifampicin drug resistance mutation reprograms macrophage metabolism through cell wall lipid changes. Nat. Microbiol..

[86] Kelly B., O’Neill L.A.J. (2015). Metabolic reprogramming in macrophages and dendritic cells in innate immunity. Cell Res..

[87] Mohamed E., Al-Khami A.A., Rodriguez P.C. (2018). The cellular metabolic landscape in the tumor milieu regulates the activity of myeloid infiltrates. Cell. Mol. Immunol..

[88] Moreira J.D.V., Hamraz M., Abolhassani M., Bigan E., Pérès S., Paulevé L., Nogueira M.L., Steyaert J.-M., Schwartz L. (2016). The redox status of cancer cells supports mechanisms behind the Warburg effect. Metabolites.

[89] Keibler M.A., Wasylenko T.M., Kelleher J.K., Iliopoulos O., Vander Heiden M.G., Stephanopoulos G. (2016). Metabolic requirements for cancer cell proliferation. Cancer Metab..

[90] Shi L., Jiang Q., Bushkin Y., Subbian S., Tyagi S. (2019). Biphasic dynamics of macrophage immunometabolism during *Mycobacterium tuberculosis* infection. MBio.

[91] Galván-Peña S., O’Neill L.A. (2014). Metabolic reprograming in macrophage polarization. Front. Immunol..

[92] Ward P.S., Thompson C.B. (2012). Metabolic reprogramming: A cancer hallmark even warburg did not anticipate. Cancer Cell.

[93] Shi L., Salamon H., Eugenin E.A., Pine R., Cooper A., Gennaro M.L. (2015). Infection with *Mycobacterium tuberculosis* induces the Warburg effect in mouse lungs. Sci. Rep..

[94] Rodríguez-Prados J.-C., Través P.G., Cuenca J., Rico D., Aragonés J., Martín-Sanz P., Cascante M., Boscá L. (2010). Substrate fate in activated macrophages: A comparison between innate, classic, and alternative activation. J. Immunol..

[95] Davies L.C., Rice C.M., McVicar D.W., Weiss J.M. (2019). Diversity and environmental adaptation of phagocytic cell metabolism. J. Leukoc. Biol..

[96] Everts B., Amiel E., van der Windt G.J., Freitas T.C., Chott R., Yarasheski K.E., Pearce E.L., Pearce E.J. (2012). Commitment to glycolysis sustains survival of NO-producing inflammatory dendritic cells. Blood.

[97] Lerner T.R., Borel S., Greenwood D.J., Repnik U., Russell M.R., Herbst S., Jones M.L., Collinson L.M., Griffiths G., Gutierrez M.G. (2017). *Mycobacterium tuberculosis* replicates within necrotic human macrophages. J. Cell Biol..

[98] Gleeson L.E., Sheedy F.J., Palsson-McDermott E.M., Triglia D., O’Leary S.M., O’Sullivan M.P., O’Neill L.A., Keane J. (2016). Cutting edge: Mycobacterium tuberculosis induces aerobic glycolysis in human alveolar macrophages that is required for control of intracellular bacillary replication. J. Immunol..

[99] Cumming B.M., Addicott K.W., Adamson J.H., Steyn A.J. (2018). *Mycobacterium tuberculosis* induces decelerated bioenergetic metabolism in human macrophages. Elife.

[100] Wang R. (2002). Two’s company, three’sa crowd: Can H2S be the third endogenous gaseous transmitter?. FASEB J..

[101] Chinta K.C., Saini V., Glasgow J.N., Mazorodze J.H., Rahman M.A., Reddy D., Lancaster J.R., Steyn A.J. (2016). The emerging role of gasotransmitters in the pathogenesis of tuberculosis. Nitric Oxide.

[102] Rahman M.A., Cumming B.M., Addicott K.W., Pacl H.T., Russell S.L., Nargan K., Naidoo T., Ramdial P.K., Adamson J.H., Wang R. (2020). Hydrogen sulfide dysregulates the immune response by suppressing central carbon metabolism to promote tuberculosis. Proc. Natl. Acad. Sci. USA.

[103] Schlesinger L. (1996). Entry of *Mycobacterium tuberculosis* into mononuclear phagocytes. Tuberculosis.

[104] Eum S.-Y., Kong J.-H., Hong M.-S., Lee Y.-J., Kim J.-H., Hwang S.-H., Cho S.-N., Via L.E., Barry C.E. (2010). Neutrophils are the predominant infected phagocytic cells in the airways of patients with active pulmonary TB. Chest.

[105] Haschek W.M., Rousseaux C., Wallig M. (2010). Chapter 6—Respiratory System. Fundamentals of Toxicologic Pathology.

[106] Lee W.L., Downey G.P. (2001). Neutrophil activation and acute lung injury. Curr. Opin. Crit. Care.

[107] Delgado-Rizo V., Martínez-Guzmán M.A., Iñiguez-Gutierrez L., García-Orozco A., Alvarado-Navarro A., Fafutis-Morris M. (2017). Neutrophil extracellular traps and its implications in inflammation: An overview. Front. Immunol..

[108] Levine A.P., Segal A.W. (2017). The NADPH oxidase and microbial killing by neutrophils, with a particular emphasis on the proposed antimicrobial role of myeloperoxidase within the phagocytic vacuole. Microbiol. Spectr..

[109] Fossati G., Moulding D.A., Spiller D.G., Moots R.J., White M.R., Edwards S.W. (2003). The mitochondrial network of human neutrophils: Role in chemotaxis, phagocytosis, respiratory burst activation, and commitment to apoptosis. J. Immunol..

[110] Van Raam B.J., Verhoeven A., Kuijpers T. (2006). Mitochondria in neutrophil apoptosis. Int. J. Hematol..

[111] Furukawa S., Saito H., Inoue T., Matsuda T., Fukatsu K., Han I., Ikeda S., Hidemura A. (2000). Supplemental glutamine augments phagocytosis and reactive oxygen intermediate production by neutrophils and monocytes from postoperative patients in vitro. Nutrition.

[112] Boxer L.A., Baehner R.L., Davis J. (1977). The effect of 2-deoxyglucose on guinea pig polymorphonuclear leukocyte phagocytosis. J. Cell. Physiol..

[113] Sica A., Strauss L. (2017). Energy metabolism drives myeloid-derived suppressor cell differentiation and functions in pathology. J. Leukoc. Biol..

[114] Groth C., Hu X., Weber R., Fleming V., Altevogt P., Utikal J., Umansky V. (2019). Immunosuppression mediated by myeloid-derived suppressor cells (MDSC) during Tumor progression. Br. J. Cancer.

[115] Hammami I., Chen J., Murschel F., Bronte V., De Crescenzo G., Jolicoeur M. (2012). Immunosuppressive activity enhances central carbon metabolism and bioenergetics in myeloid-derived suppressor cells in vitro models. BMC Cell Biol..

[116] Dibble C.C., Manning B.D. (2013). Signal integration by mTORC1 coordinates nutrient input with biosynthetic output. Nat. Cell Biol..

[117] Biswas S.K. (2015). Metabolic reprogramming of immune cells in cancer progression. Immunity.

[118] Al-Khami A.A., Zheng L., Del Valle L., Hossain F., Wyczechowska D., Zabaleta J., Sanchez M.D., Dean M.J., Rodriguez P.C., Ochoa A.C. (2017). Exogenous lipid uptake induces metabolic and functional reprogramming of tumor-associated myeloid-derived suppressor cells. OncoImmunology.

[119] McCaffrey E.F., Donato M., Keren L., Chen Z., Fitzpatrick M., Jojic V., Delmastro A., Greenwald N.F., Baranski A., Graf W. (2020). Multiplexed imaging of human tuberculosis granulomas uncovers immunoregulatory features conserved across tissue and blood. BioRxiv.

[120] Raychaudhuri B., Rayman P., Huang P., Grabowski M., Hambardzumyan D., Finke J.H., Vogelbaum M.A. (2015). Myeloid derived suppressor cell infiltration of murine and human gliomas is associated with reduction of tumor infiltrating lymphocytes. J. Neuro-Oncol..

[121] Yu J., Du W., Yan F., Wang Y., Li H., Cao S., Yu W., Shen C., Liu J., Ren X. (2013). Myeloid-derived suppressor cells suppress antitumor immune responses through IDO expression and correlate with lymph node metastasis in patients with breast cancer. J. Immunol..

[122] Korb V.C., Chuturgoon A.A., Moodley D. (2016). *Mycobacterium tuberculosis*: Manipulator of protective immunity. Int. J. Mol. Sci..

[123] Russell D.G., Cardona P.-J., Kim M.-J., Allain S., Altare F. (2009). Foamy macrophages and the progression of the human tuberculosis granuloma. Nat. Immunol..

[124] Quail D.F., Joyce J.A. (2013). Microenvironmental regulation of tumor progression and metastasis. Nat. Med..

[125] Beury D.W., Parker K.H., Nyandjo M., Sinha P., Carter K.A., Ostrand-Rosenberg S. (2014). Cross-talk among myeloid-derived suppressor cells, macrophages, and tumor cells impacts the inflammatory milieu of solid tumors. J. Leukoc. Biol..

[126] Damaghi M., Tafreshi N.K., Lloyd M.C., Sprung R., Estrella V., Wojtkowiak J.W., Morse D.L., Koomen J.M., Bui M.M., Gatenby R.A. (2015). Chronic acidosis in the Tumor microenvironment selects for overexpression of LAMP2 in the plasma membrane. Nat. Commun..

[127] Renner K., Singer K., Koehl G., Geissler E., Peter K., Siska P., Kreutz M. (2017). Metabolic hallmarks of tumor and immune cells in the tumor microenvironment. Front. Immunol..

[128] Yun J., Rago C., Cheong I., Pagliarini R., Angenendt P., Rajagopalan H., Schmidt K., Willson J.K., Markowitz S., Zhou S. (2009). Glucose deprivation contributes to the development of KRAS pathway mutations in tumor cells. Science.

[129] Cai T.-T., Ye S.-B., Liu Y.-N., He J., Chen Q.-Y., Mai H.-Q., Zhang C.-X., Cui J., Zhang X.-S., Busson P. (2017). LMP1-mediated glycolysis induces myeloid-derived suppressor cell expansion in nasopharyngeal carcinoma. PLoS Pathog..

[130] Liu G., Bi Y., Shen B., Yang H., Zhang Y., Wang X., Liu H., Lu Y., Liao J., Chen X. (2014). SIRT1 limits the function and fate of myeloid-derived suppressor cells in tumors by orchestrating HIF-1α–dependent glycolysis. Cancer Res..

[131] Ghosh H.S., McBurney M., Robbins P.D. (2010). SIRT1 negatively regulates the mammalian target of rapamycin. PLoS ONE.

[132] Belton M., Brilha S., Manavaki R., Mauri F., Nijran K., Hong Y.T., Patel N.H., Dembek M., Tezera L., Green J. (2016). Hypoxia and tissue destruction in pulmonary TB. Thorax.

[133] Tannahill G., Curtis A., Adamik J., Palsson-McDermott E., McGettrick A., Goel G., Frezza C., Bernard N., Kelly B., Foley N. (2013). Succinate is an inflammatory signal that induces IL-1β through HIF-1α. Nature.

[134] LaGory E.L., Giaccia A.J. (2016). The ever-expanding role of HIF in Tumor and stromal biology. Nat. Cell Biol..

[135] Husain Z., Huang Y., Seth P., Sukhatme V.P. (2013). Tumor-derived lactate modifies antitumor immune response: Effect on myeloid-derived suppressor cells and NK cells. J. Immunol..

[136] Hackett E.E., Charles-Messance H., O’Leary S.M., Gleeson L.E., Muñoz-Wolf N., Case S., Wedderburn A., Johnston D.G.W., Williams M.A., Smyth A. (2020). *Mycobacterium tuberculosis* Limits Host Glycolysis and IL-1β by Restriction of PFK-M via MicroRNA-21. Cell Rep..

[137] Veglia F., Tyurin V., Kagan V., Gabrilovich D. (2018). Lipids and Suppressive Functions of MDSC in Cancer.

[138] Peyron P., Vaubourgeix J., Poquet Y., Levillain F., Botanch C., Bardou F., Daffé M., Emile J.-F., Marchou B., Cardona P.-J. (2008). Foamy macrophages from tuberculous patients’ granulomas constitute a nutrient-rich reservoir for *M. tuberculosis* persistence. PLoS Pathog..

[139] Veglia F., Tyurin V., Kagan V., Gabrilovich D. (2015). Oxidized Lipids Contribute to the Suppression Function of Myeloid Derived Suppressor Cells in Cancer.

[140] Bailly C. (2020). Regulation of PD-L1 expression on cancer cells with ROS-modulating drugs. Life Sci..

[141] Corzo C.A., Cotter M.J., Cheng P., Cheng F., Kusmartsev S., Sotomayor E., Padhya T., McCaffrey T.V., McCaffrey J.C., Gabrilovich D.I. (2009). Mechanism regulating reactive oxygen species in tumor-induced myeloid-derived suppressor cells. J. Immunol..

[142] Dietlin T.A., Hofman F.M., Lund B.T., Gilmore W., Stohlman S.A., Van der Veen R.C. (2007). Mycobacteria-induced Gr-1+ subsets from distinct myeloid lineages have opposite effects on T cell expansion. J. Leukoc. Biol..

[143] Nagaraj S., Gupta K., Pisarev V., Kinarsky L., Sherman S., Kang L., Herber D.L., Schneck J., Gabrilovich D.I. (2007). Altered recognition of antigen is a mechanism of CD8+ T cell tolerance in cancer. Nat. Med..

[144] Schmielau J., Finn O.J. (2001). Activated granulocytes and granulocyte-derived hydrogen peroxide are the underlying mechanism of suppression of t-cell function in advanced cancer patients. Cancer Res..

[145] Choi H.-S., Rai P.R., Chu H.W., Cool C., Chan E.D. (2002). Analysis of nitric oxide synthase and nitrotyrosine expression in human pulmonary tuberculosis. Am. J. Respir. Crit. Care Med..

[146] Panday A., Sahoo M.K., Osorio D., Batra S. (2015). NADPH oxidases: An overview from structure to innate immunity-associated pathologies. Cell. Mol. Immunol..

[147] Cooper A.M., Segal B.H., Frank A.A., Holland S.M., Orme I.M. (2000). Transient loss of resistance to pulmonary tuberculosis in p47 ^phox−/−^ mice. Infect. Immun..

[148] Jian S.-L., Chen W.-W., Su Y.-C., Su Y.-W., Chuang T.-H., Hsu S.-C., Huang L.-R. (2017). Glycolysis regulates the expansion of myeloid-derived suppressor cells in tumor-bearing hosts through prevention of ROS-mediated apoptosis. Cell Death Dis..

[149] Satoh H., Moriguchi T., Taguchi K., Takai J., Maher J.M., Suzuki T., Winnard P.T., Raman V., Ebina M., Nukiwa T. (2010). Nrf2-deficiency creates a responsive microenvironment for metastasis to the lung. Carcinogenesis.

[150] Ohl K., Fragoulis A., Klemm P., Baumeister J., Klock W., Verjans E., Böll S., Möllmann J., Lehrke M., Costa I. (2018). Nrf2 is a central regulator of metabolic reprogramming of myeloid-derived suppressor cells in steady state and sepsis. Front. Immunol..

[151] Ohl K., Tenbrock K. (2018). Reactive oxygen species as regulators of MDSC-mediated immune suppression. Front. Immunol..

[152] Corzo C.A., Condamine T., Lu L., Cotter M.J., Youn J.-I., Cheng P., Cho H.-I., Celis E., Quiceno D.G., Padhya T. (2010). HIF-1α regulates function and differentiation of myeloid-derived suppressor cells in the tumor microenvironment. J. Exp. Med..

[153] Gabrilovich D.I., Velders M.P., Sotomayor E.M., Kast W.M. (2001). Mechanism of immune dysfunction in cancer mediated by immature Gr-1+ myeloid cells. J. Immunol..

[154] Drabczyk-Pluta M., Werner T., Hoffmann D., Leng Q., Chen L., Dittmer U., Zelinskyy G. (2017). Granulocytic myeloid-derived suppressor cells suppress virus-specific CD8(+) T cell responses during acute Friend retrovirus infection. Retrovirology.

[155] Lei G.-S., Zhang C., Lee C.-H. (2015). Myeloid-derived suppressor cells impair alveolar macrophages through PD-1 receptor ligation during Pneumocystis pneumonia. Infect. Immun..

[156] Poe S.L., Arora M., Oriss T.B., Yarlagadda M., Isse K., Khare A., Levy D.E., Lee J.S., Mallampalli R.K., Chan Y.R. (2013). STAT1-regulated lung MDSC-like cells produce IL-10 and efferocytose apoptotic neutrophils with relevance in resolution of bacterial pneumonia. Mucosal Immunol..

[157] Sammicheli S., Kuka M., Di Lucia P., de Oya N.J., De Giovanni M., Fioravanti J., Cristofani C., Maganuco C.G., Fallet B., Ganzer L. (2016). Inflammatory monocytes hinder antiviral B cell responses. Sci. Immunol..

[158] Fooksman D.R., Nussenzweig M.C., Dustin M.L. (2014). Myeloid cells limit production of antibody-secreting cells after immunization in the lymph node. J. Immunol..

[159] Lelis F.J., Jaufmann J., Singh A., Fromm K., Teschner A.C., Pöschel S., Schäfer I., Beer-Hammer S., Rieber N., Hartl D. (2017). Myeloid-derived suppressor cells modulate B-cell responses. Immunol. Lett..

[160] Rastad J.L., Green W.R. (2016). Myeloid-derived suppressor cells in murine AIDS inhibit B-cell responses in part via soluble mediators including reactive oxygen and nitrogen species, and TGF-β. Virology.

[161] Park M.J., Lee S.H., Kim E.K., Lee E.J., Park S.H., Kwok S.K., Cho M.L. (2016). Myeloid-derived suppressor cells induce the expansion of regulatory B cells and ameliorate autoimmunity in the sanroque mouse model of systemic lupus erythematosus. Arthritis Rheumatol..

[162] Crook K.R., Jin M., Weeks M.F., Rampersad R.R., Baldi R.M., Glekas A.S., Shen Y., Esserman D.A., Little P., Schwartz T.A. (2015). Myeloid-derived suppressor cells regulate T cell and B cell responses during autoimmune disease. J. Leukoc. Biol..

[163] Pålsson-McDermott E.M., O’Neill L.A. (2020). Targeting immunometabolism as an anti-inflammatory strategy. Cell Res..

[164] Du Plessis N., Kotze L.A., Leukes V., Walzl G. (2018). Translational Potential of Therapeutics Targeting Regulatory Myeloid Cells in Tuberculosis. Front. Cell. Infect. Microbiol..

[165] Li L., Wang L., Li J., Fan Z., Yang L., Zhang Z., Zhang C., Yue D., Qin G., Zhang T. (2018). Metformin-induced reduction of CD39 and CD73 blocks myeloid-derived suppressor cell activity in patients with ovarian cancer. Cancer Res..

[166] Qin G., Lian J., Huang L., Zhao Q., Liu S., Zhang Z., Chen X., Yue D., Li L., Li F. (2018). Metformin blocks myeloid-derived suppressor cell accumulation through AMPK-DACH1-CXCL1 axis. Oncoimmunology.

[167] Singhal A., Jie L., Kumar P., Hong G.S., Leow M.K.-S., Paleja B., Tsenova L., Kurepina N., Chen J., Zolezzi F. (2014). Metformin as adjunct antituberculosis therapy. Sci. Transl. Med..

[168] Condamine T., Dominguez G.A., Youn J.-I., Kossenkov A.V., Mony S., Alicea-Torres K., Tcyganov E., Hashimoto A., Nefedova Y., Lin C. (2016). Lectin-type oxidized LDL receptor-1 distinguishes population of human polymorphonuclear myeloid-derived suppressor cells in cancer patients. Sci. Immunol..

